# Levels and confounders of morning cortisol collected from adolescents in a naturalistic (school) setting

**DOI:** 10.1016/j.psyneuen.2008.06.010

**Published:** 2008-10

**Authors:** Shona J. Kelly, Robert Young, Helen Sweeting, Joachim E. Fischer, Patrick West

**Affiliations:** a Division of Epidemiology and Public Health, University of Nottingham, Queen's Medical Centre, Nottingham NG7 2RD, UK; b MRC Social & Public Health Sciences Unit, 4 Lilybank Gardens, Glasgow G12 8RZ, UK; c Mannheim Institute of Public Health, Mannheim Medical Faculty, University of Heidelberg, Ludolf-Krehl-Strasse 7-11, D-68167 Mannheim, Germany

**Keywords:** Hydrocortisone, Adolescent, Saliva, Stress, Cross-sectional studies, Social environment

## Abstract

Salivary cortisol is widely used in research but little is known about the typical, or expected, functioning of the HPA-axis in adolescents in naturalistic settings, nor whether the extensive array of confounders documented in the literature is applicable in this situation.

In a school-based study, 2995 15-year-old pupils provided two saliva samples, 30 min apart, in morning sessions timed to capture peak cortisol decline. The collection protocol was a balance between the large sample size obtainable in a school situation and a limited number of samples, constrained by the school timetable. In addition, pupils completed a questionnaire containing items previously shown to be associated with cortisol levels (e.g. time since awakening and life events), and their height and weight were measured. Outcome measures were cortisol levels at Times 1 and 2, and change (per minute) in cortisol between the two time points.

Median (IQR) cortisol levels for males and females were 10.5 (8.1) and 11.6 (9.3) nmol/L at Time 1, and 8.2 (6.0) and 8.1 (6.5) nmol/L at Time 2. 73% had a decline in cortisol level of more than 10% across the two time points, compatible with the expected diurnal pattern. In bivariate analyses, cortisol sampled on Monday, times of measurement and since awakening, prior smoking and several life events were associated with cortisol levels at Times 1 and 2 in both sexes. However, in multivariate analysis, few of these variables remained after controlling for times of measurement and since awakening and, in addition, the final models differed between the sexes. Two events (friend dying and splitting with a boy/girlfriend) predicted cortisol levels in both sexes while age, maturity, recent eating and smoking were predictors only in males. Several factors associated with cortisol change differed from those observed for absolute levels. Further adjustment for school clustering affected some associations, particularly time of measurement.

This study managed many of the problems found in naturalistic research on cortisol and provides norms for morning cortisol levels in 15-year-old adolescents.

## 1 Introduction

With the advent of its measurement in saliva, cortisol has become widely used in research on stress. However, with a few notable exceptions (e.g. Rosmalen et al., 2005; Power and Hertzman, 2006) studies of cortisol have been conducted on relatively small samples, often including fewer than 100 individuals. In addition, there are little data on whether measured levels are within the expected (i.e. ‘normal’) range for community-living people (Patel et al., 2004). Despite a number of studies in relation to cortisol and adolescent depression, anxiety or chronic fatigue (e.g. Goodyer et al., 1998), it has been suggested that “little is known about the typical or expected functioning of the HPA-axis in adolescents. This lack of research on cortisol activity in normal adolescents in their everyday environments is surprising” (Adam, 2006).

While the assessment of salivary cortisol has been well validated (e.g. Kirschbaum and Hellhammer, 2000), and is suitable for large-scale studies (Rosmalen et al., 2005), it is complicated by a quirk of cortisol secretion; namely, that in addition to responding to stressors, cortisol follows a daily circadian rhythm in most people. The lowest levels generally occur around midnight and usually begin to rise before waking, continuing for 30–45 min thereafter (Schmidt-Reinwald et al., 1999; Wust et al., 2000), with a daily peak between 05:00 h and 08:00 h. This is followed by a rapid decline for the next few hours, then a gradual decline over the remainder of the day (Kirschbaum and Hellhammer, 1989). Although one approach to this variation within naturalistic settings is to collect repeated measures and use the mean, this is very crude and fails to capture changes in the daily circadian rhythm (Hruschka et al., 2005). A range of more sophisticated methods have therefore been used to characterise this, including indices of the slope of the decline, assessments in the morning and evening, and area under the curve measures (Ranjit et al., 2005).

Previous studies, focusing on either natural decline or reactivity, have identified a number of factors likely to impact on cortisol levels collected in the field setting. Firstly, because of its circadian pattern, time of day and time since awakening have a significant effect on measured levels (Kudielka and Kirschbaum, 2003). Secondly, studies have found the morning pattern to differ between males and females. Compared with men, women show a delayed decrease (Pruessner et al., 1997), and have higher morning levels (Steptoe et al., 2000). Morning salivary cortisol levels have also been found to be higher in girls, particularly post-pubertally, than boys (Netherton et al., 2004; Rosmalen et al., 2005). A third factor is day of the week, in respect of which there have been inconsistent findings. Thus, while some studies of working adults have found increased cortisol excretion (Maina et al., 2007) on work compared with weekend days, others have found no differences (Vidovic et al., 2007). A fourth possible factor is age and body size. However, although there is some evidence of positive associations between cortisol levels and age, body mass and pubertal stage in children and adolescents (Tornhage, 2002), results are inconclusive (Rosmalen et al., 2005; Netherton et al., 2004). In addition, there are a number of activities and states which have been found to impact on cortisol levels. These include the use of corticosteroid medication (Ricciardolo, 2007), eating (Rosmond et al., 2000), caffeine in dietary doses (Lovallo et al., 2005), smoking (Kirschbaum et al., 1992; Steptoe and Ussher, 2006), exercise (Ben-Aryeh et al., 1989; Filaire et al., 2001; Urhausen et al., 1995), and the experience of aggression, stress, distress and negative emotion (Haller et al., 2005; Adam, 2006). Serious physical illness also results in increased cortisol levels (Van den Berghe et al., 1998). Finally, levels are increased among those reporting work strain or more general stress (Steptoe et al., 2000), although results in respect of the relationship between life events and cortisol are inconsistent. Thus, while some studies find life events to be associated with higher cortisol levels (Strickland et al., 2002), others find no relationship (Goodyer et al., 1998).

Against this background, this paper is based on a study of 2995 adolescents (age 15), who provided two samples in a school setting, approximately 30 min apart, starting around 09.00 h, thus capturing the period of most rapid daytime decline. The aims are: (1) to present salivary cortisol levels among a large sample of community-living adolescents; and (2) to examine differences in respect of factors previously found to be associated with salivary cortisol measures. In addition, we describe our methodology for collecting samples in a naturalistic environment.

## 2 Methods

### 2.1 Study participants and procedures

Data are drawn from a survey conducted among pupils within 22 schools situated in the Central Clydeside Conurbation, a predominantly urban area in and around Glasgow city in the West of Scotland. The study received approval from the relevant Glasgow University Ethics Committee, participating local authorities and schools. The sampling scheme aimed to obtain a representative sample by selecting schools within strata based on geographical location (within Glasgow City or not), religious status (Catholic/Non-denominational) and deprivation (represented by proportion of pupils in receipt of a clothing grant). Subsequent analyses found that participating schools did not differ significantly from the remainder in the Central Clydeside Conurbation in respect of these dimensions, nor for total pupil roll or exam achievement by the end of statutory schooling (Sweeting et al., 2008). Within selected schools, all pupils in (Scottish) Secondary 4, the final statutory year of schooling, were invited to participate via letters sent to parents, including opt-out consent forms. In addition, participating pupils positively consented to all measures.

During the school-based sessions, which took place during the first morning class (beginning around 09.00 h and lasting 45–55 min), pupils filled in a questionnaire, completed a brief interview, had their height and weight measured and provided two salivary cortisol samples. The study was well resourced with a survey team to pupil ratio of one to seven.

The total sample comprised 3194 15-year olds (representing 81% of the eligible sample of 3950) who filled in a questionnaire, of whom 3057 had their height and weight measured (the discrepancy is accounted for by pupils absent from school on the survey day who completed and returned their questionnaires by post). Of those who completed questionnaires, 185 failed to provide one or both cortisol samples, 13 were extreme values presumed to be contaminated with blood and excluded from further analysis, and one additional pair of samples collected 1 h later than the rest of the sample was also eliminated. The resulting 2995 pupils (94% of those who filled in a questionnaire, and 76% of the eligible sample) with two useable cortisol samples were included in the analyses presented here; the mean age of this sample was 15 years 5 months (S.D. 3.8 months).

Pupils completing a questionnaire were more likely than non-responders to be female, from less deprived areas and from schools with fewer ethnic minority pupils. Among questionnaire completers, those providing cortisol samples were more likely to be male, from less deprived areas, from schools with more ethnic minority pupils, less likely to have recently moved school, to be a smoker, have used cannabis or experienced victimisation. Probabilistic weights have been derived to compensate for this (Sweeting et al., 2008).

### 2.2 Salivary cortisol measurement and assay

Cortisol was obtained using the Salivette sampling device (Sarstedt, 51582 Numbrecht, Germany); a centrifuge tube with a suspended insert containing a sterile neutral cotton wool swab. At the beginning of the survey, pupils were instructed not to eat or drink during the session, and to remove chewing gum. They were provided with two pre-labelled Salivettes, and around 5 min into questionnaire completion, the whole group was instructed to remove and chew on the cotton wool for approximately 2 min, after which completed samples were collected by the survey team. This process was repeated half an hour later, around 5 min before the end of the session (mean (S.D.) time between samples = 31.3 (3.7) min). Saliva samples were stored within the MRC Social Public Health Sciences Unit at −20 °C and sent in batches, via international courier, to the laboratory (Institute for Behavioural Sciences, Swiss Federal Institute of Technology, Zurich) for analysis.

Samples were analysed by single analysis using the Tekan roboting pipetting system (Genesis 150, Tekan, Stäfa, Switzerland) using the IBL luminometric assay (IBL, Hamburg, Germany) according to the specifications of the manufacturer at the laboratory of the Institute of Behavioral Sciences (Swiss Federal Institute of Technology, Zurich, Switzerland). Approximately 10% of samples were run in duplicate for assessment of inter-assay (all values < 8%) and intra-assay reliability (all values < 5%). Samples outside the detection range of the assay were repeated in appropriate dilutions and, in addition, some of the most extreme were reanalysed in another laboratory (C. Kirschbaum, Dept. of Psychology, Technical University, Dresden, Germany).

### 2.3 Other measures

Physical confounders: Preliminary analyses of the relationship between cortisol levels and day of the week showed, as might be expected, that levels differed for Monday, compared with Tuesday–Friday. Day of the week is therefore represented by the dichotomous variable Monday versus other weekdays. Time of each cortisol sample is measured in hours, centred on the mean, thus distinguishing those measured earlier compared with those measured later. Time since awakening was derived from the difference between self-reported awakening and time at first cortisol sample; mean (S.D.) = 1.87 (0.61) h. Age was calculated in months based on date of birth as provided by the pupils. Body Mass Index (BMI) was calculated from height (m) and weight (kg), measured by the research interviewers using Leicester portable stadiometers and Seca scales, and categorized as ‘normal’, ‘overweight’, and ‘obese’ using IOTF sex specific age-cut offs (Cole et al., 2000). A fourth category, ‘underweight’ was defined using a similar method, using cut-offs based on the WHO-defined grade 3 adult thinness of BMI less than 18.5 kg/m^2^ (Cole et al., 2007). Physical maturity was evaluated by the survey team, who classified pupils as below average (for age and sex), about average and above average.

Other known confounders: The questionnaire included seven factors previously shown in the literature to affect levels: whether the respondent took asthma medication or used an inhaler; had a cold; and in the previous hour had eaten anything, drunk coffee, smoked a cigarette, exercised for more than 10 min, or had a fight or argument lasting more than 20 min.

Life events: Pupils were also presented with a list of life events, closely based on those used in other studies of children and adolescents (e.g. Coddington, 1972; Sweeting and West, 1994). This asked if they had experienced any of the following events within the previous year: a serious accident, illness/injury in a close family member, parental separation or divorce, death of a close family member, being attacked/hurt, death of a close friend, parental job loss, changed schools, moved house, parent has new live-in partner, or done badly in an important exam. In addition, they were asked if, within the previous month, they had been in trouble with the police or gone to court; their parents had a serious row; they had been in serious trouble at school; or they had broken up with a girlfriend/boyfriend. Individual life events were evaluated for their association with cortisol levels as were counts of the total number of previous year, previous month and all events.

Self-rated stress during survey session: Pupils completed two self-reports of perceived stress, one at the beginning of the questionnaire and the second at the end, in which they were asked to select from a 7-step Likert scale anchored with 1 = ‘completely stressed’ and 7 = ‘totally relaxed’.

### 2.4 Data analysis

Cortisol was examined as absolute levels at Times 1 and 2, and the change in cortisol between the two samples (nmol/L/min). All absolute cortisol levels were logged for analysis; because of the skewed nature of the data, medians and interquartile ranges (IQR) are reported (Tables 1a and 1b).

Bivariate screening analyses of the relationship between the three cortisol measures and the potential confounders and life events were conducted via *t*- or *F*-tests for categorical, and Pearson's correlations for continuous, measures. For the multivariate analyses, stepwise backwards regression was conducted in three separate blocks. Block one entered all physical confounders which had been identified as significant at *p* ≤ .10 in the preceding bivariate screening analyses. Block two entered all variables remaining as significant at *p* ≤ .05 in block one, plus all variables representing other known confounders identified as significant (*p* ≤ .10) in the bivariate screening analyses, and block three entered all variables remaining as significant (*p* ≤ .05) in block two, plus bivariately significant (*p* ≤ .10) individual life events. Finally block three was repeated, entering total previous year and month life events. The final multivariate model retained only variables conventionally significant (*p* ≤ .05). As the pupils were clustered in schools we used the Huber–White sandwich estimator (SPSS v. 15) to adjust for clustering. Results are reported with, and without, this adjustment.

Finally, since the results of analyses conducted with weighted data (see earlier) were not different, here we present those based on unweighted data.

## 3 Results

Median (IQR) cortisol levels for males and females were 10.5 (8.1) and 11.6 (9.3) nmol/L at Time 1 (T1), and 8.2 (6.0) and 8.1 (6.5) nmol/L at Time 2 (T2), representing a change (decline) per minute of 0.07 (0.16) and 0.10 (0.15). Sex differences in respect of the T1 levels and the change per minute were significant (*t* = −4.9, *p* ≤ .001; *t* = −5.6, *p* ≤ .001). Further analyses (not shown) found that overall 27% (males = 32%, females = 22%, *χ*^2^ = 42.1, *p* < .001) had either an increase or no change (±10%) in cortisol levels between T1 and T2, but the majority (73%) had a decline of more than 10% across the two cortisol measures, suggesting that most did not find the survey stressful. Consistent with this, there was no indication of increased self-rated stress from T1 to T2; mean scores were 5.4 at both times among males, and changed only very slightly, from 5.1 at T1 to 5.0 at T2 among females. Comparison of those whose cortisol levels increased or showed no change with those where it declined found no differences in sample collection time, nor were those with an increasing or constant level more likely to have only recently arisen; indeed, this group had been awake 9 and 6 min, respectively, longer on average than those whose levels declined across the two samples.

Correlations between T1 and T2 cortisol levels were high (males = .733, females = .810), as were those between levels at T1 and change over time (males = .557, females = .592), higher initial levels associated with a steeper decline. There was also a weak, but significant, positive association between change over time and cortisol levels at T2 for females only (males = −.039, females = .113).

Tables 1a (males) and 1b (females) show associations, significant at ≥.10, between the three cortisol measures and the potential confounders and life events. In males, the factors associated with cortisol levels at T1 and T2 were similar; times of measurement and since awakening and recent eating (negative), and cortisol sampled on Monday, age, maturity rating, having recently smoked a cigarette, death of a friend and new parental partner in the previous year, splitting with a girlfriend in the previous month, and the total year's and previous month's life events scores (positive). Recent exercise (negative), experience of an attack in the previous year, and trouble with the police in the previous month (positive) were significantly associated with cortisol levels at T1 only. Poor exam performance in the previous year was associated with increasing T2 levels.

In females, there was a similar negative association between cortisol at both T1 and T2 and times of measurement and since awakening, and positive associations with cortisol sampled on Monday, recent smoking, death of a friend, serious parental row, splitting with a boyfriend and the total life events scores. In addition, recent eating was negatively associated with both measures. Finally, increasing levels at T1 were associated with previous year parental separation and job loss, and at T2 with obesity, a current cold, previous year change of school and previous month trouble with the police.

In males, change per minute in cortisol levels between the two collections was associated negatively with time since awakening and recent exercise, and positively with underweight, recent smoking and fight or argument, a number of specific life events and the total life events scores. For females, there were fewer associations with change per minute, but sampled on Monday, times of measurement and since awakening and two of the individual life events were significant.

We found no significant associations between either cortisol levels or change in cortisol for asthma medication, recent coffee consumption, previous year family illness or moved house, or serious trouble at school in the previous month.

In multivariate analyses, entering the potential confounders in blocks, the final models for both males and females in respect of cortisol at T1 and T2 included cortisol sampled on Monday, times of measurement and since awakening and previous month split with a girl/boyfriend (see Table 2). In addition, the models for males included age, maturity, recent eating and smoking, and in the T2 model death of a friend; for females, the only other variable included was death of a friend. In analyses (not shown), entering total life events (year and month), rather than individual events, the T1 final models for both males and females retained total year events, while the T2 final model for males retained neither total year nor month events, but that for females retained total month events. Far fewer variables were included in the final models for change per minute between T1 and T2; the model for males included time since awakening and attacked or hurt in the previous year (or total year events), while that for females included only sampled on a Monday and time since awakening.

After adjusting for possible clustering effects the final models, with a few exceptions, had virtually identical beta estimates, but the significance levels were marginally altered. Although the results were broadly the same, there was some evidence that the effect of a few predictors on cortisol levels, particularly, as might be expected, time of measurement, could be attributable to clustering by school.

## 4 Discussion

We have described a school-based method for obtaining salivary cortisol from a large sample of adolescents which provides an alternative to the restriction of lab-based fixed-time collection methods. Although this was conducted at little extra cost or effort over that of a questionnaire-based survey, apart from the purchase of Salivettes and laboratory analysis of the samples, very few other studies have adopted such a method (although see Lupien et al., 2000). Provision of samples in the presence of peers, researchers and survey staff may have increased compliance and adherence to our protocols, and immediate collection meant that samples were not lost due to incorrectly sealing the Salivette.

There is evidence that compliance with the regime is poor among community living individuals who are instructed to collect samples throughout the day (Kudielka et al., 2003). By using a monitored collection protocol and recording the time of awakening and collection, we have eliminated this problem. Set against this is the fact that although, in order to address both the diurnal cortisol rhythm and intra-individual variability, the ideal protocol would be multiple saliva collections throughout a day, repeated over several days. However, this would not have been acceptable to the schools participating in the study.

Salivary cortisol levels in this adolescent sample appear consistent with those of other studies; median levels of 10.5 nmol/L (male) and 11.6 nmol/L (female) at Time 1, on average 1 h 52 min after awakening. Previous studies have reported median salivary cortisol levels (nmol/L) of 9.7 (male) and 10.2 (female) in a sample of 32 15-year-old Swedish pupils who provided samples around 08:30 h (Tornhage and Alfven, 2006), and 21.3 (male) and 18.9 (female) among 248 UK 16–98 year olds who provided wake-up samples (Patel et al., 2004). Mean levels of 6.3 have been reported among 94 Iranian 6–14-year olds who provided samples at 08.00 h (Safarzadeh et al., 2005); 15.4 among 1768 Dutch 10–12-year olds 30 min after awakening (Rosmalen et al., 2005); and 21.0 (male) and 21.9 (female) among 6470 UK 45-year olds 45 min after awakening (Power et al., 2006).

Generally, research on cortisol tends to focus on two different perspectives, which are sometimes combined. First, a wealth of research exists regarding the salivary cortisol secretion patterns following a brief exposure to a psychosocial or physical stress. In this perspective, possible cortisol increases are expected to be observable about 15–45 min after the onset of the stressor. Second, other researchers have attempted to elucidate fluctuations in circadian secretion patterns in response to the chronic stress of daily living (Kelly and Hertzman, 2001). There is evidence of an increase in levels across stressful events such as examinations (Martinek et al., 2003), suggesting the possibility that pupils might have perceived our questionnaire, brief interview and measurement of height and weight as stressful. The fact that self-reported stress remained the same, at the ‘relaxed’ end of the scale, and that 73% of participants had a decline in cortisol levels between T1 and T2, suggests that this was not the case.

Although the protocol was specifically designed to avoid the awakening response, our two measurements appear to have captured a portion of the decline from this peak. Currently there is a dearth of field studies specifically elucidating the normal shape of the circadian cortisol secretion later than the first hour after awakening in adolescents. The majority of protocols of studies in adults take two or more samples during the first hour after awakening, but subsequent samples are spaced later in the morning. One of the few datasets that examined early morning levels was a study in critical care nurses, in whom a decline of 20–30% during the second hour after awakening was observed (Fischer et al., 2000).

The factors identified by previous studies as associated with increased levels were supported to some extent. The T1 level was higher among females, as was the decline, resulting in no sex difference by T2. Steptoe et al. (2000) found a similar pattern in a study of adults, with higher levels among women at 08:00–08:30 h, no sex difference at 10:00–10:30 h and higher levels among men at 12:00–12:30 h. Netherton et al. (2004), who also found higher levels in females (particularly post-pubertally) suggest a sex divergence in HPA functioning, associated with puberty. We also found fewer factors to be associated with cortisol levels or change in levels among females. This may have arisen because much of the previous work on adolescents has focused on comparing groups with and without specific problems or health conditions, a methodology which enhances any differences. Our representative population sample may have failed to capture what are, in general, subtle differences between extremes.

Day of the week, time of measurement and time since awakening all have a significant impact on levels and should always be included in analysis. We found little effect of age on cortisol levels, but had a very narrow age range. Rosmalen et al. (2005), with a similarly restricted range (10–12 years), also found no age effect. They note that findings about age and cortisol response are inconsistent, and suggest that repeated measurements in a longitudinal cohort study are required. Despite no association with age, there was a difference in respect of interviewer's maturity rating, cortisol being lower among males rated ‘below average’. The association was attenuated somewhat when the analysis was adjusted for clustering within schools. Also, these ratings were crude, and may have been made on the basis of body size and shape or other features. Previous results relating to cortisol levels and BMI or pubertal stage have been inconsistent (e.g. Netherton et al., 2004; Rosmalen et al., 2005.)

Of the confounders suggested by previous literature, current cold, recent eating, cigarette smoking and exercise were related to one or more of the measures, asthma medication or an inhaler and recent coffee to none. Coffee was reported by only 3% of the sample, so only a very strong effect would result in significant differences in cortisol levels between the groups. Eleven percent reported asthma medication, however it is possible that a large proportion of this group took it only infrequently.

Total previous year and month life events were associated in bivariate analysis with levels at both T1 and T2 and, among males, with change per minute. Total year events were retained in the final multivariate T1 model for both males and females. Previous studies have been most consistent in finding associations between cortisol levels and recent or immediate events or hassles. Of the individual events, the two which showed the most consistent relationship were death of a friend in the previous year and splitting with a girl or boyfriend in the previous month. The first of these suggests the salience of an unexpected death. The second highlights the importance, in mid-adolescence, of an event signifying loss or peer rejection (Coleman, 1974; Hendry et al., 1996). The fact that the effects were attenuated in some of the models correcting for clustering within schools is interesting and suggests the possibility of school level effects. In a subsequent paper we will explore the relationship between cortisol and the social milieu of the adolescents in greater depth. Our results suggest that social factors/events may only have a significant effect on cortisol levels in particular environments.

This study has managed many of the problems found in naturalistic research of cortisol levels, such as timing of sample collection. Having more samples collected across the day would have been preferred but is difficult to achieve in a school setting. In addition, the narrow age range and school-based sample may mean that those who are most likely to be exposed to cortisol secretion-altering circumstances are under-represented.

In summary, this paper has provided insights into daily cortisol secretion and, even with a limited number of samples, has highlighted the importance of accounting for time since awakening time of sampling and day of the week when collecting samples in a naturalistic setting. We have also demonstrated, with a large sample size, that very few of the published list of confounders are important after controlling for these time factors.

## Role of funding source

Funding for this study was provided by the UK Medical Research Council (MRC) as part of the Youth and Health Programme (WBS U.1300.00.007) at the Social and Public Health Sciences Unit. The MRC had no role in the study design, data collection, analysis or interpretation, nor in the writing of this paper or the decision to submit it for publication.

## Conflict of interest

The authors have no actual or potential conflict of interest including any financial, personal or other relationships with other people of organizations within the previous 3 years of beginning the work that could inappropriately influence, or be perceived to influence, our work.

## Figures and Tables

**Table 1a:** Bivariate associations (significant at .10 or less) with confounders, life events, and psychosocial measures by sex—males

	Cortisol—Time 1					Cortisol—Time 2					Per minute change T1–T2				
	Median	(IntQ range)	*R*	*t/F*	*p*	Median	(IntQ range)	*R*	*t/F*	*p*	Median	(IntQ range)	*R*	*t/F*	*p*
	10.5	(8.1)				8.2	(6.0)				.07	(.16)			
Physical confounds															
Day of week															
Monday	13.6	(8.5)				10.3	(7.2)								
Tuesday–Friday	10.0	(7.6)		*7.8*	*<.001*	7.9	(5.5)		*7.6*	*<.001*					

Time of measurement (hours—centred on mean)			*−.197*		*<.001*			*−.204*		*<.001*					
Time since woken (hours)			*−.276*		*<.001*			*−.146*		*<.001*			*−.213*		*<.001*
Age (months)			*.088*		*.001*			*.088*		*.001*					

BMI															
Underweight											.08	(.16)			
Normal											.08	(.15)			
Overweight											.05	(.16)			
Obese											.05	(.19)		*2.7*	*.042*

Maturity rating															
Below average	9.3	(7.8)				7.0	(5.7)								
Average	10.6	(8.0)				8.3	(6.2)								
Above average	10.7	(8.3)		*3.4*	*.032*	8.6	(5.6)		*8.2*	*<.001*					

Misc confounds															
Eaten (hour)															
Yes	10.5	(7.3)				8.0	(5.6)								
No	10.6	(9.3)		*−1.8*	*.077*	9.0	(6.9)		*−3.6*	*<.001*					

Cigarette (hour)															
Yes	11.9	(9.2)				9.5	(7.7)				.10	(.20)			
No	10.4	(7.8)		*3.3*	*.001*	8.2	(5.8)		*2.9*	*.003*	.07	(.16)		*1.8*	*.074*

Exercise (hour)															
Yes	10.0	(6.9)									.05	(.16)			
No	10.7	(8.3)		*−2.1*	*.039*						.07	(.15)		*−2.8*	*.005*

Fight or argument (hour)															
Yes											.12	(.18)			
No											.07	(.16)		*1.7*	*.094*

Life events															
Accident (year)															
Yes											.10	(.16)			
No											.07	(.16)		*2.4*	*.015*

Family death (year)															
Yes											.08	(.15)			
No											.06	(.16)		*1.9*	*.063*

Attacked or hurt (year)															
Yes	11.4	(8.8)									.09	(.17)			
No	10.3	(7.9)		*2.5*	*.014*						.07	(.15)		*2.8*	*.005*

Friend died (year)															
Yes	13.0	(9.0)				10.5	(9.3)								
No	10.4	(7.9)		*3.3*	*.001*	8.2	(5.8)		*3.7*	*<.001*					

New parental partner (year)															
Yes	12.1	(8.0)				9.2	(7.4)								
No	10.4	(8.1)		*2.3*	*.023*	8.2	(5.9)		*1.7*	*.093*					

Poor exam performance (year)															
Yes						8.4	(6.3)								
No						8.0	(5.8)		*2.1*	*.033*					

Trouble with police (month)															
Yes	11.4	(8.4)									.10	(.15)			
No	10.3	(8.0)		*2.5*	*.013*						.06	(.16)		*2.6*	*.009*

Spilt with girl/boyfriend (month)															
Yes	11.9	(9.8)				9.0	(7.4)								
No	10.4	(7.7)		*2.8*	*.005*	8.1	(5.8)		*2.7*	*.006*					

Sum total life events			*.106*		*<.001*			*.074*		*.005*			*.090*		*.001*
Sum last year events			*.096*		*<.001*			*.064*		*.015*			*.082*		*.002*
Sum last month events			*.088*		*.001*			*.062*		*.017*			*.074*		*.005*

*Notes*: All correlations (*R*), *t*-tests (*t*) and ANOVAs (*F*) performed on cortisol T1 and T2 use logged data. Time of measurement = time of cortisol 1 (Cortisol—Time 1 and per minute change T1 − T2); time of cortisol 2 (Cortisol—Time 2). No significant associations were found for males between any of the cortisol measures and asthma medication, cold (current), previous hour coffee, previous year family illness, parental separation, parental job loss, new school or moved house, nor previous month serious parental row or serious trouble at school.

**Table 1b:** Bivariate associations (significant at .10 or less) with confounders, life events, and psychosocial measures by sex—females

	Cortisol—Time 1					Cortisol—Time 2					Per minute change T1–T2				
	Median	(IntQ range)	*R*	*t/F*	*p*	Median	(IntQ range)	*R*	*t/F*	*p*	Median	(IntQ range)	*R*	*t/F*	*p*
	11.6	(9.3)				8.1	(6.5)				.10	(.15)			
Physical confounds															
Day of week															
Monday	15.5	(10.4)				10.9	(7.7)				.14	(.17)			
Tuesday–Friday	11.0	(8.6)		*9.5*	*<.001*	7.7	(5.9)		*8.5*	*<.001*	.09	(.15)		*4.0*	*<.001*

Time of measurement (hours—centred on mean)			*−.231*		*<.001*			*−.188*		*<.001*			*−.069*		*.007*
Time since woken (hours)			*−.295*		*<.001*			*−.239*		*<.001*			*−.186*		*<.001*

BMI															
Underweight						7.2	(5.2)								
Normal						8.0	(6.5)								
Overweight						8.3	(6.3)								
Obese						8.7	(8.5)		*2.4*	*.066*					

Misc confounds															
Current cold															
Yes						8.5	(6.4)								
No						8.0	(6.3)		*2.3*	*.023*					

Eaten (hour)															
Yes	11.4	(9.0)				8.0	(6.1)								
No	11.9	(9.8)		*−2.0*	*.041*	8.5	(7.0)		*−2.0*	*.050*					

Cigarette (hour)															
Yes	12.9	(8.9)				8.9	(6.2)								
No	11.5	(9.4)		*2.9*	*.004*	8.0	(6.5)		*2.5*	*.011*					

Life events															
Parental separation															
Yes	12.8	(8.0)													
No	11.5	(9.4)		*2.1*	*.035*										

Family death (year)															
Yes											.10	(.15)			
No											.10	(.15)		*1.7*	*.096*

Friend died (year)															
Yes	16.1	(12.4)				11.5	(9.9)								
No	11.5	(9.0)		*4.7*	*<.001*	8.0	(6.2)		*4.3*	*<.001*					

Parental job loss															
Yes	13.0	(9.8)													
No	8.0	(6.3)		*1.7*	*.094*										
New School (year)															
Yes						7.2	(6.9)								
No						8.1	(6.4)		*−1.7*	*.083*					
New parental partner (year)															
Yes											.09	(.12)			
No											.10	(.15)		*−1.7*	*.091*

Trouble with police (month)															
Yes						9.1	(5.7)								
No						8.0	(6.5)		*1.8*	*.081*					

Serious parental row															
Yes	11.9	(9.3)				8.0	(7.1)								
No	11.5	(9.3)		*2.0*	*.047*	8.1	(6.3)		*1.9*	*.055*					

Spilt with girl/boyfriend (month)															
Yes	12.9	(9.9)				9.1	(7.7)								
No	11.4	(9.0)		*3.2*	*.001*	7.9	(6.2)		*4.0*	*<.001*					

Sum total life events			*.069*		*.008*			*.079*		*.002*					
Sum last year events			*.051*		*.049*			*.053*		*.040*					
Sum last month events			*.072*		*.005*			*.094*		*<.001*					

*Notes*: All correlations (*R*), *t*-tests (*t*) and ANOVAs (*F*) performed on cortisol T1 and T2 use logged data. Time of measurement = time of cortisol 1 (Cortisol—Time 1 and per minute change T1 − T2); time of cortisol 2 (Cortisol—Time 2). No significant associations were found for females between any of the cortisol measures and age, maturity rating, asthma medication, previous hour coffee, exercise, or fight/argument, previous year accident, family illness, attacked/hurt, moved house or poor exam performance, nor previous month serious trouble at school.

**Table 2:** Final multivariate results with and without adjustment for clustering by school (those without adjustment significant at .05 or less)

	Logged cortisol—Time 1						Logged cortisol—Time 2						Per minute change T1 − T2					
	No adjustment for clustering			Adjusted for clustering			No adjustment for clustering			Adjusted for clustering			No adjustment for clustering			Adjusted for clustering		
	*B*	S.E.	*p*	*B*	S.E.	*p*	*B*	S.E.	*p*	*B*	S.E.	*p*	*B*	S.E.	*p*	*B*	S.E.	*p*
Males																		
Physical confounds (Block 1)																		
Cortisol on Monday	.094	.015	*<.001*	.093	.036	*.017*	.103	.015	*<.001*	.102	.042	*.025*						
Time of measurement (hours)	−.194	.033	*<.001*	−.196	.102	*.069*	−.235	.040	*<.001*	−.238	.133	*.087*						
Time since woken (hours)	−.085	.009	*<.001*	−.086	.014	*<.001*	−.038	.009	*<.001*	−.039	.011	*.002*	−.054	.007	*<.001*	−.054	.008	*<.001*
Age (months)	.004	.001	*.014*	.004	.002	*.054*	.003	.002	*.033*	.003	.002	*.132*						

Maturity rating																		
Average																		
Below average	−.044	.017	*.009*	−.044	.021	*.051*	−.061	.017	*<.001*	−.061	.020	*.006*						
Above average	−.017	.015	*.247*	−.017	.008	*.051*	−.002	.015	*.915*	.000	.015	*.986*						

Misc confounds (Block 2)																		
Eaten (hour)	−.026	.012	*.030*	−.026	.016	*.124*	−.047	.012	*<.001*	−.047	.017	*.014*						
Cigarette (hour)	.062	.023	*.008*	.061	.023	*.015*	.047	.024	*.047*	.048	.025	*.073*						

Life events (Block 3)																		
Attacked or hurt (year)													.036	.014	*.008*	.036	.012	*.007*
Friend died (year)							.051	.025	*.046*	.050	.032	*.134*						
Spilt with girl/boyfriend (month)	.050	.020	*.011*	.051	.023	*.036*	.043	.020	*.034*	.042	.023	*.086*						

Females																		
Physical confounds																		
Cortisol on Monday	.120	.015	*<.001*	.122	.026	*<.001*	.111	.015	*<.001*	.111	.025	*<.001*	.045	.011	*<.001*	.047	.016	*.008*
Time of measurement (hours)	−.189	.033	*<.001*	−.187	.088	*.045*	−.173	.039	*<.001*	−.170	.119	*.170*						
Time since woken (hours)	−.117	.011	*<.001*	−.118	.015	*<.001*	−.094	.011	*<.001*	−.094	.014	*<.001*	−.054	.008	*<.001*	−.055	.009	*<.001*

Life events																		
Friend died (year)	.090	.024	*<.001*	.089	.032	*.012*	.097	.025	*<.001*	.096	.029	*.003*						
Spilt with girl/boyfriend (month)	.044	.015	*.004*	.042	.016	*.017*	.055	.015	*<.001*	.054	.018	*.008*						

*Notes*: Analyses performed on cortisol T1 and T2 use logged data. Time of measurement = time of cortisol 1 (Cortisol—Time 1 and per minute change T1 − T2); time of cortisol 2 (Cortisol—Time 2).
